# Mood Lability Induced by Pallidal Deep Brain Stimulation in a Patient with Meige Syndrome

**DOI:** 10.1002/mdc3.14332

**Published:** 2025-01-21

**Authors:** Kai Grimm, Alessandro Gulberti, Wolfgang Hamel, Monika Pötter‐Nerger, Carsten Buhmann, Christian K.E. Moll, Simone Zittel

**Affiliations:** ^1^ Department of Neurology University Medical Center Hamburg‐Eppendorf Hamburg Germany; ^2^ Department of Neurosurgery University Medical Center Hamburg‐Eppendorf Hamburg Germany; ^3^ Department of Neurophysiology and Pathophysiology University Medical Center Hamburg‐Eppendorf Hamburg Germany

Meige syndrome is a segmental dystonia that involves blepharospasm and oromandibular dystonia. When botulinum toxin and oral medication fail to alleviate symptoms, deep brain stimulation (DBS) of the globus pallidus internus (GPi) may be considered as treatment. Known side effects of GPi‐DBS include bradykinesia, speech impairment and phosphenes.^1^

We present a patient that first visited the local outpatient clinic when she was 67 years old. She had been suffering from involuntary squinting and mouth movements for 6 years. Blepharospasm and oromandibular muscle contractions with platysma involvement led movement disorder experts (CB, MPN, SZ) to diagnose Meige syndrome. There was no history of previous psychiatric disorders or intake of antipsychotic medication. Brain magnetic resonance imaging was unremarkable. After 5 years, inefficacy of botulinum toxin injections and oral medication resulted in bilateral DBS electrode implantation in the GPi (Medtronic Activa PC, lead model 3389). 3‐D reconstruction of electrode position with computed tomography (CT) and the LEAD‐DBS toolbox^2^ revealed regular placement of the leads (Fig. 1A–C): center of left/right ventral‐most contacts relative to the mid‐commissural point (MCP) *x* = 20.3/18.9 lateral to midline; *y* = 0.2/0.4 anterior to MCP; *z* = 7.4/5.7 inferior to AC‐PC level. Before surgery, the patient scored 22 points on the Burke‐Fahn‐Marsden Dystonia Rating Scale (BFMDRS) and 5 points on the Beck Depression Inventory (BDI‐II).

**Figure 1:**
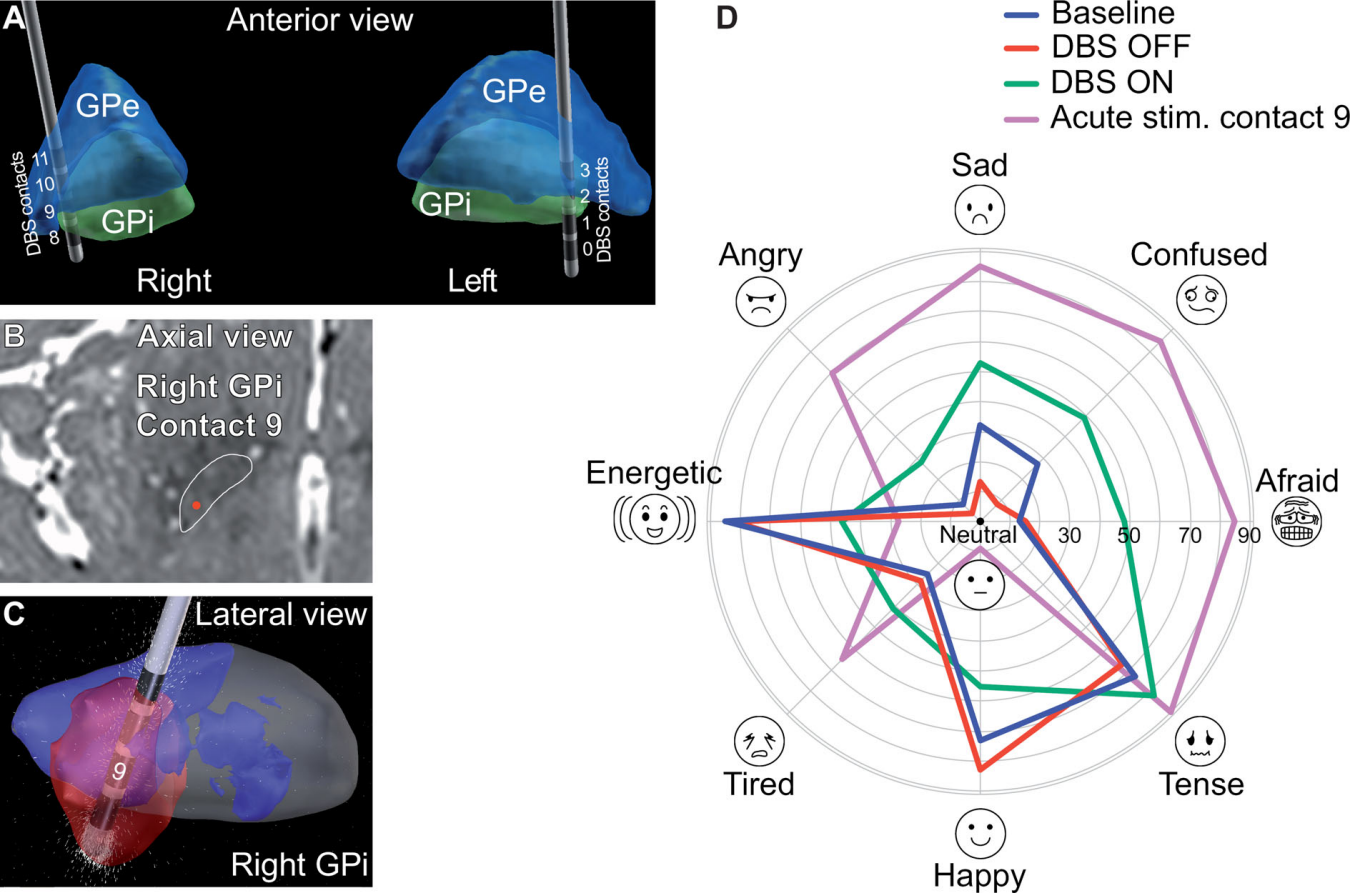
DBS lead location and effects on the VAMS. (A) Lead positions in MNI space. (B) Postoperative CT fused with preoperative MRI; red dot: contact 9. (C) Volume of tissue activated (red). (D) Emotional states of the VAMS across DBS conditions. DBS, deep brain stimulation; VAMS, Visual Analog Mood Scales.

After surgery, dystonic symptoms improved, but the patient experienced mood lability and frequent episodes of crying that severely affected her quality of life (QoL). To investigate this unusual side effect, we assessed the patient three times over a three‐week period, each week applying a different DBS mode. The patient entered the evaluation with the left GPi electrode delivering stimulation and the right GPi electrode essentially turned off (baseline). At the second and third visit, DBS was turned off on both sides (DBS‐OFF) and on (DBS‐ON: GPi left: 1−/2+, 3.0 V, 90 μs, 130 Hz; GPi right: 9−/10+, 1.0 V, 90 μs, 130 Hz; see Fig. 1) for 1 week, respectively. DBS‐ON was a stimulation mode expected to induce moderate mood changes in the patient while also improving motor symptoms. The order of DBS‐ON/‐OFF was randomized and the rater (KG) and the patient were blinded to the stimulation condition. At each visit, dystonia motor symptom severity with BFMDRS, QoL with the Craniocervical Dystonia Questionnaire (CDQ‐24), and mood with the Hospital Anxiety and Depression Scale (HADS) as well as the Visual Analog Mood Scales (VAMS) were assessed. During the first visit, a systematic contact testing up to the side effect threshold was additionally performed. Side effects were noted and, after 1 min at side effect threshold intensity, the *sad* subscale of VAMS was sampled.

The test results for the three visits and DBS conditions are presented in Fig. 1D and Supplement 1. In summary, motor symptom severity remained virtually unchanged. QoL and mood scores in the DBS‐ON condition worsened compared to baseline and improved in the DBS‐OFF condition. Electrode contact testing revealed a marked increase in VAMS‐sad from 32 to 68 points with stimulation of contact 9 (right GPi) at the side effect threshold of 3.7 V. Stimulation of this contact led to sudden crying (see Video 1). Stimulation of the other contacts was not associated with any changes in mood or crying.

**Video 1:** The video demonstrates the acute effects of right globus pallidus internus (GPi) stimulation in the patient, including sudden mood lability and crying induced by GPi‐DBS. The stimulation parameters were: contact 9‐/IPG+, intensity 3.8 V, pulse width 60 μs, frequency 130 Hz.

To summarize, we observed unusual side effects characterized by sudden depressive mood swings induced by GPi‐DBS in this Meige syndrome patient. These mood alterations were immediate and consistently associated with the activation of the second‐most ventral contact of the right GPi electrode. Notably, while motor symptom improvement due to DBS was about 50%, no substantial change in motor symptoms was observed during the double‐blind investigation after 1 week in the DBS‐ON and ‐OFF condition. This lack of change may be attributed to neuroplastic long‐term effects resulting from chronic GPi‐DBS^3^ or placebo effects.

Clinically significant, acute‐onset negative affective changes due to GPi‐DBS are very rare with only one similar case in Parkinson's disease having been reported before.^4^ Most studies of mood alterations following GPi‐DBS reported no significant effects or improvements.^5^, ^6^ Okun carried out systematic electrode contact testing to assess acute mood fluctuations using the VAMS in a cohort of nine Parkinson's disease patients treated with STN or GPi‐DBS.^7^ Among the four GPi‐DBS patients, two showed improvements on several VAMS subscales. One patient reported minor worsening which affected only the *tense* subscale of VAMS.

One possible reason for the described mood changes due to DBS may be current spread to the amygdalofugal tract. This phenomenon has previously been reported in a patient with ventral dislocation of the GPi electrode.^8^ However, in the present case, electrode position was regular, making this mechanism less likely. An alternative consideration is the aberrant functional connectivity of the GPi with limbic structures, which may be found in patients with Meige's syndrome.^9^, ^10^

The present case highlights the need to carefully evaluate not only motor but also psychiatric non‐motor features in dystonia patients after GPi‐DBS.

## Author Roles

(1) Research Project: A. Conception, B. Organization, C. Execution. (2) Data Analysis: A. Design, B. Execution, C. Review and Critique. (3) Manuscript Preparation: A. Writing of the First Draft, B. Review and Critique. All authors have contributed to, seen and approved the manuscript.

K.G.: 1A, 1B, 1C, 2A, 2B, 3A.

A.G.: 1A, 1B, 1C, 2A, 2B, 3A.

W.H.: 2C, 3B.

M.P.: 2C, 3B.

C.B.: 2C, 3B.

C.M.: 2B, 2C, 3B.

S.Z.: 1A, 2A, 2C, 3B.

## Disclosures

**Ethical Compliance Statement:** This study was conducted in agreement with the declaration of Helsinki (1967). It should be noted that this was not a clinical trial but rather a case report, and the patient was treated in accordance with standard clinical practice.

**Funding Sources and Conflict of Interest:** No specific funding was received for this work. The authors declare that there are no conflicts of interest relevant to this work.

**Financial Disclosures for the previous 12 months:** KG and CB declare that there are no additional disclosures to report. AG has been reimbursed for travel expenses from Medtronic. WH received lecture fees and honoraria for serving on advisory boards, travel grants and educational grants from Abbott, Aleva, Boston Scientific, and Medtronic. MP‐N received personal study fees from Abbvie, Abbott, Boston Scientific, Medtronic, Bial, Desitin and Zambon. CM received funding from the German Research Foundation (CRC 936, C8), received consulting fees and honoraria from Inomed and is on the advisory board of Inomed. SZ received honoraria from Merz Pharma and grant funding from the German Research Foundation, the European Commission, the Damp Foundation and the Arbeitskreis Botulinumtoxin.

## Declaration of patient consent

The patient provided her informed written consent to participate in the double‐blind, randomized design investigation, granting permission for access to her clinical records, the recording and utilization of video materials, as well as the writing and publishing of the case report.

## Supporting information


**TABLE S1.** Data from clinical scores were gathered across three sessions that took place a week apart. The order of DBS‐ON and DBS‐OFF was randomized, and both the patient and the rating physician were blinded to the DBS condition. DBS, deep brain stimulation; BFMDRS, Burke‐Fahn‐Marsden Dystonia Rating Scale; CDQ‐24, Craniocervical Dystonia Questionnaire; HADS, Hospital Anxiety and Depression Scale; VAMS, Visual Analog Mood Scales.

## Data Availability

The data that support the findings of this study are available from the corresponding author upon reasonable request.
