# Supplementary material for: Mood Lability Induced by Pallidal Deep Brain Stimulation in a Patient with Meige Syndrome
Source: Mov Disord Clin Pract. 2025 Jan 21;12(5):704–6. doi: 10.1002/mdc3.14332 (PMC12070168; doi:10.1002/mdc3.14332)
Supplement: Supplementary file 1 — TABLE S1. Data from clinical scores were gathered across three sessions that took place a week apart. The order of DBS‐ON and DBS‐OFF was randomized, and both the patient and the rating physician were blinded to the DBS condition. DBS, deep brain stimulation; BFMDRS, Burke‐Fahn‐Marsden Dystonia Rating Scale; CDQ‐24, Craniocervical Dystonia Questionnaire; HADS, Hospital Anxiety and Depression Scale; VAMS, Visual Analog Mood Scales. [file MDC3-12-704-s001.docx]

**Supplementary Table 1.** Data from clinical scores were gathered across three sessions that took place a week apart. The order of DBS-ON and DBS-OFF was randomized, and both the patient and the rating physician were blinded to the DBS condition. DBS: deep brain stimulation, BFMDRS: Burke-Fahn-Marsden Dystonia Rating Scale, CDQ-24: Craniocervical Dystonia Questionnaire, HADS: Hospital Anxiety and Depression Scale, VAMS: Visual Analog Mood Scales.

|  | BASELINE | DBS-OFF | DBS-ON |
| --- | --- | --- | --- |
| DBS parameters: right GPi | 9-/10+, 0.1 V, 60 µs, 130 Hz | off | 9-/10+, 1.0 V, 90 µs, 130 Hz |
| DBS parameters: left GPi | 1-/2+, 3.0 V, 60 µs, 130 Hz | off | 1-/2+, 3.0 V, 90 µs, 130 Hz |
| BFMDRS | 11.5 | 12.0 | 12.5 |
| CDQ-24 | 25; max score: stigma (13) | 17; max score: stigma (11) | 37; max score: stigma (17) |
| HADS (depression) | 6 | 3 | 8 |
| HADS (anxiety) | 4 | 3 | 3 |
| VAMS (sad) | 32 | 13 | 53 |
| VAMS (happy) | 73 | 83 | 55 |
